# Editorial: The Reach of War in Childhood: Ukrainian Children Beyond the Frontlines and the Imperative to Scale and Strengthen Mental Health Services for All

**DOI:** 10.1016/j.jaacop.2026.06.005

**Published:** 2026-06-24

**Authors:** María Alejandra Gutiérrez-Torres, Theresa S. Betancourt

**Affiliations:** a Research Program on Children and Adversity, Boston College School of Social Work, Chestnut Hill, Massachusetts

## Abstract

In 2024, UNICEF (United Nations Children’s Fund) estimated that more than 473 million children, approximately 1 in 6 globally, lived in areas affected by armed conflict, setting new records.^1^ Rightfully so, a great deal of the international response has focused on the mental health needs of children closest to active combat. However, the study by Lestarevic Stojkov and Lero *et al.*^2^ in this issue of *JAACAP Open* contributes to a growing body of evidence showing that we need to look far beyond the front lines when it comes to understanding and responding to the mental health needs of children affected by war. In a school-based sample of 4,753 children aged 7 to 17 in Ternopil, a western Ukrainian city distant from the front lines, 40.3% screened positive for significant posttraumatic stress symptoms on the 8-item Child Revised Impact of Events Scale (CRIES-8). Whereas children near active conflict zones undoubtedly face the greatest risks, the Ternopil findings remind us that mental health impacts of war extend beyond what humanitarian frameworks typically address, and effective responses should focus on strengthening mental health supports for all children affected by war.

In 2024, UNICEF (United Nations Children’s Fund) estimated that more than 473 million children, approximately 1 in 6 globally, lived in areas affected by armed conflict, setting new records.^1^ Rightfully so, a great deal of the international response has focused on the mental health needs of children closest to active combat. However, the study by Lestarevic Stojkov and Lero *et al.*^2^ in this issue of *JAACAP Open* contributes to a growing body of evidence showing that we need to look far beyond the front lines when it comes to understanding and responding to the mental health needs of children affected by war. In a school-based sample of 4,753 children aged 7 to 17 in Ternopil, a western Ukrainian city distant from the front lines, 40.3% screened positive for significant posttraumatic stress symptoms on the 8-item Child Revised Impact of Events Scale (CRIES-8). Whereas children near active conflict zones undoubtedly face the greatest risks, the Ternopil findings remind us that mental health impacts of war extend beyond what humanitarian frameworks typically address, and effective responses should focus on strengthening mental health supports for all children affected by war.

Lestarevic Stojkov and Lero *et al.*^2^ conducted a cross-sectional survey as part of the preintervention assessment in a cluster randomized trial of Teaching Recovery Techniques Plus Parenting (TRT+P). Using an opt-out consent method across 26 schools, the authors collected CRIES-8 self-reports from children, along with demographic information from 1,512 caregivers and 1,815 teachers. Three key findings emerged. First, the prevalence of significant posttraumatic stress disorder symptoms (40.3%) exceeded the 35% found in a recent national study using the Child and Adolescent Trauma Screen^3^ and was much higher than the 1.2% reported in war-unaffected Kirovohrad in 2016.^4^ Second, girls reported higher intrusion, avoidance, and total scores than boys (44.2% vs 36.0%). Third, children aged 7 to 10 had the highest scores, a pattern that differs from that observed in CRIES-8 results in non-war settings.

These results should be considered alongside the study’s acknowledged methodological limitations. CRIES-8 is a brief, nonspecific screening tool that does not include an events questionnaire asking about particular types of trauma. Thus, children were not asked to endorse war-related trauma events, so the symptoms identified cannot be definitively linked to the conflict.^5^ The lack of a specific war-related events questionnaire documenting indirect exposures, such as displacement, parental conscription, or proximity to bombardments, is a significant limitation. Data on exposure to trauma events require careful ethical planning, but are also useful for understanding different pathways that contribute to higher symptom levels. Indeed, research in other war-affected settings has critiqued the focus only on war-related events, highlighting that daily hardships, often made much worse by war, have a significant role to play in the mental health distress of children and families in war zones. In addition, from a developmental perspective, cross-sectional research that captures only a snapshot in time has limitations in understanding factors shaping risk and resilience over time. Much more longitudinal research is needed in understanding the mental health of children affected by war and intergenerational dynamics of war in a range of cultures and settings.^6^ Although these limitations do not negate the importance of this study, its role is best seen as a preliminary step that highlights, rather than provides answers to, the underlying mechanisms and developmental processes involved.

For children in Ternopil, though seldom exposed to direct bombardment, their experiences of air-raid sirens, disrupted school routines, pervasive media coverage, parental conscription, fears of family separation, economic instability, and grief within extended kinship networks were anecdotally high. Such daily stressors are not secondary to the war’s impact; rather, they are a key pathway through which conflict affects children and help explain why even a city far from the front lines might show such high prevalence figures of mental health stressors.^7^ These findings align with the recent BMJ Collection on Child Mental Health in Conflict Settings, which highlights that children in fragile and conflict-affected areas rarely face isolated traumatic events. Instead, they endure interconnected, ongoing stressors, such as direct violence, displacement, poverty, family separation, disrupted services, and compromised caregiving environments. These factors collectively influence mental health and development over the lifespan of children and increasingly across generations.^8^

The age gradient noted by Lestarevic Stojkov and Lero *et al.*^2^ (in which younger children fare worse) is consistent with how war affects caregiving environments. Younger children are more dependent on their caregivers for emotional regulation, and their sense of safety is closely linked to attachment relationships, which war quietly disrupts. Eltanamly *et al.*^9^ analyzed 38 quantitative studies on war-affected families and found that war exposure interrupts responsive parenting and that such disruption in parenting is associated with poorer child adjustment. The higher intrusion symptoms observed in girls may, in part, be due to gender-specific vulnerabilities in conflict zones, such as increased exposure to and fear of sexual violence, a concern highlighted in research on adolescent girls in war.^10^ Overall, these findings emphasize that the family is not only a setting, but also a key pathway through which war impacts children and a critical point for intervention.

This is the moment for the field to focus its efforts. Three decades after the first 1995 United Nations Report of the Impact of Armed Conflict on Children led by former First Lady of Mozambique, Graça Machel, put war-affected children on the international agenda, the evidence base for culturally and contextually adapted, effective mental health and psychosocial support (MHPSS) interventions has expanded significantly. However, implementation remains the main obstacle: most evidence-based MHPSS interventions reach only a small portion of children in need and are not well integrated into existing national systems.^11^ Moving forward, a phased approach is needed to integrate evidence-based intervention packages into existing platforms that already serve children in war-affected settings, such as primary care, schools, and child and social protection services. This should be combined with workforce development, sustainable funding, and meaningful community involvement in both design and delivery.^11^ Additionally, MHPSS efforts should be embedded within the humanitarian, development, and peace sectors to ensure that emergency responses are linked to long-term recovery and system strengthening.^7^ The Ukrainian response, including that led by First Lady Olena Zelenska’s national MHPSS program that integrates mental health into schools and primary care, provides an early example of large-scale integration.^7^

It is impressive that this cross-sectional study is not a stand-alone effort. The study by Lestarevic Stojkov and Lero *et al.*^2^ is part of the initial phase of an intervention trial: children identified as needing support will, by design, receive it. This reflects a principle that should be standard in research on effects of conflict: no survey should be conducted without ensuring that services are available. The 40.3% of children meeting criteria for significant posttraumatic stress disorder should be viewed not just as an isolated result, but rather as a starting point and a reminder that the critical question for our field is not whether war impacts children far from the front lines. It does. The real question is whether the systems created to support these children, both directly and indirectly affected, will progress beyond pilot studies and prevalence surveys to develop and strengthen ongoing, integrated, evidence-based MHPSS services to respond to the developmental and longer-term needs of children affected by war. The field has advanced, and Ukraine is positioned to demonstrate the emergence of a new era of MHPSS response for children and families in war-affected settings where a longer-term view on MHPSS services is taken and efforts are made to scale evidence-based solutions with quality, local ownership, and sustainability in every context where children carry the burden of war.

## CRediT authorship contribution statement

**María Alejandra Gutiérrez-Torres:** Conceptualization, Writing – original draft, Writing – review & editing. **Theresa S. Betancourt:** Conceptualization, Writing – original draft, Writing – review & editing.
